# Whole Brain Measurement of Amyloid Plaques and Neuroinflammation AD Model Mice

**DOI:** 10.1002/alz70859_103712

**Published:** 2025-12-25

**Authors:** Nathaniel Guanzon, Yessenia Gallegos, Chase Redd, Erik Castillo, Eric Blaes, Ricardo Azevedo, Sunil Gandhi, Damian G. Wheeler

**Affiliations:** ^1^ Translucence Biosystems, Inc, Irvine, CA USA; ^2^ Center for the Neurobiology of Learning and Memory, Department of Neurobiology and Behavior, University of California, Irvine, Irvine, CA USA; ^3^ Activity Signaling, LLC, San Diego, CA USA

## Abstract

**Background:**

Traditional histological methods have long been fundamental to neuroscience research. However, imaging deep into tissues has historically required slicing and mounting on slides, limiting observations to predefined regions of interest. Recent advances in optical clearing and light sheet imaging have opened an exciting new avenue for brain‐wide, cellular resolution immunostaining at the forefront of a dimensional shift from 2D to 3D histology. One area where these methods have particular utility is in the development of CNS therapeutics where they can be used to examine brain‐wide target engagement and phenotypic efficacy. Toward this end, we have developed methodologies for tissue clearing and AI‐powered quantification of Amyloid plaques and microglia and are disseminating this technology to the basic research and drug discovery community.

**Methods:**

Using our iDISCO‐based tissue clearing kits and a light sheet microscope, we can image micron‐scale resolution immunoreactivity across entire intact mouse brains. Further, our Translucence Teravoxel Toolkit (3TK) software identifies individual immunostained cells and objects throughout the brain and registers them to a 3D atlas to produce an unbiased, regionalized read‐out of cellular patterns across 100’s brain areas.

**Results:**

Using antibodies targeting the microglial protein Iba1, we have developed multiple workflows for brain‐wide segmentation of microglia, providing regional metrics of shape and microglial activation. To validate our 3D tissue clearing methods, in LPS‐treated brains we also performed traditional 2D slice IHC. In AD model 5xFAD mice, we demonstrated that our methods can also label Amyloid plaques throughout the intact brain, allowing for automated brain‐wide quantification. In these mice, rather than displaying the ramified morphology seen in WT mice, microglia become condensed and colocalized with β‐Amyloid plaques. We developed ML‐powered workflows quantify these plaque‐associated microglia (PAMs) and found that PAM levels throughout the brain correlated with regional densities of Amyloid plaques.

**Conclusion:**

This technology is now being disseminated with our BRAIN Initiative‐funded iDISCO‐based tissue clearing kits and cloud‐based quantification pipeline, enabling neuroscientists to easily employ next generation 3D immunohistochemistry for unbiased, complete, and anatomically precise mapping of the efficacy of CNS therapeutics affecting amyloid deposition and neuroinflammation.

We thank NIMH for Grant R43MH122070.

